# Adenine Nucleotides Attenuate Murine T Cell Activation Induced by Concanavalin A or T Cell Receptor Stimulation

**DOI:** 10.3389/fphar.2017.00986

**Published:** 2018-01-10

**Authors:** Yuria Shinohara, Mitsutoshi Tsukimoto

**Affiliations:** Department of Radiation Biosciences, Faculty of Pharmaceutical Sciences, Tokyo University of Science, Chiba, Japan

**Keywords:** ATP, T cell, purinergic receptor, inflammation, IL-2, concanavalin A, T cell receptor

## Abstract

Extracellular ATP and its metabolites affect various cellular immune responses, including T cell function, but there are apparently conflicting reports concerning the effects of adenine nucleotides on T cells. For example, it has been reported that ATP-mediated activation of P2 receptor is involved in T cell activation; activation of adenosine receptors suppresses T cell function; and 1 mM ATP induces T cell death via activation of P2X7 receptor. Therefore, in this work we investigated in detail the effects of 100–250 μM ATP, ADP, or AMP on murine T cell activation. First, an *in vitro* study showed that pretreatment of murine splenic T cells with 100–250 μM ATP, ADP, or AMP significantly suppressed the concanavalin A (ConA)-induced release of cytokines, including IL-2. This suppression was not due to induction of cell death via the P2X7 receptor or to an immunosuppressive effect of adenosine. ATP attenuated the expression of CD25, and decreased the cell proliferation ability of activated T cells. The release of IL-2 by ConA-stimulated lymphocytes was suppressed by post-treatment with ATP, as well as by pretreatment. These results suggest that exogenous ATP suppresses the activation of T cells. Secondly, we evaluated the effect of ATP in a ConA-treated mice. Treatment with ATP attenuated the increase of IL-2 concentration in the blood. Overall, these results suggest that adenine nucleotides might have potential as supplemental therapeutic agents for T cell-mediated immune diseases, by suppressing T cell activation.

## Introduction

Activation of T cells by MAPKs induces various responses necessary for immune function. There are three major MAPK pathways that operate in T-cells (Rincon et al., 2000a,b; Gong et al., 2001), involving c-Jun NH2-terminal kinase, extracellular signal regulatory protein kinase (ERK) (Fischer et al., 2005; Zhang and Dong, 2005), and p38 MAPK. Activation of these MAPKs results in nuclear translocation and promoter binding of transcription factors, and results in gene expression of a number of mediators involved in the inflammatory response (Marshall et al., 1995; Whitehurst and Geppert, 1996). Elevated intracellular Ca^2+^ ion concentration is also essential for T cell activation. In lymphocytes, calcium release-activated calcium channels open in response to calcium depletion of the endoplasmic reticulum and increase the intracellular Ca^2+^ concentration (Oh-hora, 2009), thereby activating expression of numerous cytokine genes (Gallo et al., 2006). In particular, the production of IL-2 and the induction of CD25 (IL-2 receptor α chain) expression (Kondo et al., 1993; Nelson et al., 1994) are involved in T cell activation and proliferation (Nelson et al., 1994; Lin and Leonard, 1997). Pathological activation of T cells plays an important role in autoimmune diseases, including multiple sclerosis, rheumatoid arthritis, and autoimmune hepatitis.

Concanavalin A (ConA) is a plant lectin isolated from *Canavalia ensiformis* (jack bean) seeds that binds to various glycosyl proteins and to α-D-mannose residues on glycolipids. It induces mitogenic activity of T lymphocytes and has various bioactivities (Lei and Chang, 2009). Treatment of mice with ConA increased production of inflammatory cytokines such as IL-2, IL-4, IL-6, IL-10, IL-12, TNF-α, and IFN-γ (Sass et al., 2002).

ATP is released from various types of cells into the extracellular compartment. ATP and its metabolites, such as ATP, ADP, AMP and adenosine, regulate various physiological effects via the ligand-gated ion channel P2X receptor and the metabotropic G protein-coupled P2Y receptor (Burnstock, 2009; Burnstock and Boeynaems, 2014). Previous studies have demonstrated that P2X, P2Y, and adenosine receptors play roles in both TCR stimulation-induced and hypertonic stress-induced T cell activation (Yip et al., 2007; Woehrle et al., 2010). Many researchers, including our group, have reported involvement of extracellular ATP and purinergic receptors in these actions, showing that extracellular ATP induces T cell activation via P2X7, P2X4, and P2Y6 receptors (Schenk et al., 2008; Tsukimoto et al., 2009; Yip et al., 2009; Tokunaga et al., 2010; Woehrle et al., 2010). However, the inhibitory effect of ATP and its metabolites on T cell activation is still not completely understood. It was reported that activation of P2X7 receptor by ATP (0.5–1 mM) induces T cell death (Chused et al., 1996; Tsukimoto et al., 2006). On the other hand, 250 nM ATP induces T cell proliferation, cytokine release, and molecular adhesion (Trabanelli et al., 2012). Another report indicated that ATP suppresses T cell proliferation (Weiler et al., 2016). That is, the functions of ATP appear to depend on its concentration. Thus, a detailed understanding of the effects of exogenously added adenine nucleotides on T cells is very important for the elucidation of T cell functions. Although adenosine is well known to inhibit T cell function via activation of adenosine receptor, it is poorly soluble in water, whereas ATP is very soluble, and shows very low cytotoxicity. Thus, if ATP can suppress T cell activation, sustained intravenous injection of ATP in patients with immune disease or graft-versus-host disease might be a promising candidate for supplemental treatment of their disease.

In this study, we found that activation of murine T cells is suppressed by ATP, as well as by its metabolites, ADP and AMP. ATP inhibited the production of inflammatory cytokine IL-2 at both the mRNA and protein levels, as well as expression of CD25 and activation-associated T cell proliferation. In addition, intravenous administration of ATP into mice suppressed ConA-induced elevation of serum IL-2 level in mice. These results suggest that combination of ATP with existing treatment modalities might be beneficial in patients with T cell mediated-immune diseases.

## Materials and Methods

### Reagents

Concanavalin A (ConA) were purchased from Sigma–Aldrich. Anti-CD3ε mAb was purchased from R&D Systems (United States). Anti-CD28 mAb was from eBioscience (United States). ATP, ADP, and adenosine were from Sigma (United States). PPADS, BDBD, MRS2578, SCH442416, PSB36, MRS3777, A438079, CGS15943, PSB603, and MRS2111 were from Tocris Bioscience (United Kingdom). Suramin and oxATP were purchased from Sigma–Aldrich. NF449 was from Abcam (United Kingdom). Adenosine 5′-[γ-thio]triphosphate tetralithium salt (ATP-γ-S), α,β-methyleneadenosine 5′-triphosphate lithium salt (α,β-Me-ATP), BzATP, α,β-methyleneadenosine 5′-diphosphate sodium salt (α,β-Me-ADP), and 2-methylthioadenosine diphosphate trisodium salt (2-MeS-ADP) were purchased from Sigma–Aldrich. Anti-ERK1/2 mAb and anti-phospho-ERK 1/2 (Thr^202^/Tyr^204^) mAb and other secondary Abs were obtained from Cell Signaling Technology (United States). All other chemicals used were of the highest purity available.

### Animals

Male BALB/c mice (21.6 ± 1.04 g) were purchased from Sankyo Labo Service (Japan) and used at 6 weeks of age. They were housed in plastic cages with paper chip bedding and bred in rooms kept at a temperature of 23 ± 2°C and a relative humidity of 55 ± 10% under a 12 h light–dark cycle. They were allowed free access to tap water and normal diet, CE-2 (CLEA Co. Ltd.). The mice were treated and handled according to the Guiding Principles for the Care and Use of Laboratory Animals of the Japanese Pharmacological Society and with the approval of Tokyo University of Science’s Institutional Animal Care and Use Committee (permit numbers S17007 and S17010).

### Preparation of Splenic Lymphocytes

Splenocytes were isolated from the spleen of BALB/c mice, and were purified by means of hemolysis. Cells were washed twice with complete RPMI1640 medium and re-suspended in RPMI1640-based buffer (Tsukimoto et al., 2006). To remove adherent cells such as macrophages and dendritic cells, splenocytes were incubated for 1.5 h in a plastic cell culture plate for 1 h in an atmosphere of 5% CO_2_/95% air at 37°C. Non-adherent cells, mainly lymphocytes, were collected and washed once with RPMI1640-based buffer.

### Cytokine Production

Splenocytes (6.0 × 10^6^ cells/well) were stimulated with ConA (5 μg/mL) in a 96-well cell culture plate in RPMI 1640 medium containing 10% heat-inactivated FBS, 100 units/ml of penicillin, and 100 μg/ml of streptomycin in an atmosphere of 5% CO_2_, 95% air at 37°C. For TCR stimulation, splenocytes were incubated with anti-CD28 mAb (0.5 mg/ml) (eBioscience, San Diego, CA, United States) in a 96-well plastic cell culture plate, coated with anti-CD3ε mAb (5 μg/ml) (R&D Systems, Minneapolis, MN, United States), in RPMI1640 medium containing 10% FBS in an atmosphere of 5% CO_2_/95% air at 37°C. After incubation, the culture supernatant was harvested for determination of IL-2 and IL-6. The concentrations of IL-2, IL-6, and IL-17 were measured by means of ELISA as described below. Wells of a 96-well plate were coated with purified anti-mouse IL-2 (1:500) (eBioscience) and IL-6 (1:500) (eBioscience) IL-17 mAb. The plate was incubated overnight at 4°C, then washed with PBS containing 0.05% Tween-20, and non-specific binding was blocked with PBS containing 1% BSA for 1 h at room temperature. The plate was washed again, and the culture supernatant was added. After 2 h at room temperature, the plate was washed again, and biotin-conjugated anti-mouse IL-2 (1:1000) (eBioscience) and IL-6 (1:500) (eBioscience) IL-17 mAb were added for 1 h at room temperature. The plate was further washed, and Streptavidin, Peroxidase Conjugated, Solution (Wako) was added. The plate was washed, 3,3′,5,5′-tetramethylbenzidine was added, and incubation was continued for several minutes. The reaction was stopped by adding 5 N H_2_SO_4_. The absorbance at 450 nm (contrast wavelength: 570 nm) was measured. Standard curves were established with recombinant mouse IL-2 (31–2000 pg/mL), IL-6 (31–2000 pg/mL), or IL-17 (31–2000 pg/mL), and the concentrations of IL-2 and IL-6 were estimated from the standard curves. IFN-γ, IL-4, TNF-α production was determined with mouse IFN-γ, IL-4, TNF-α Quantikine ELISA kits (R&D Systems) according to the manufacturer’s instructions.

### Evaluation of Cell Damage

Cell damage was quantified in terms of released LDH activity (Tsukimoto et al., 2006). Splenocytes were incubated with adenine nucleotides for 1 h, and the culture supernatants were collected. Release of LDH into the cell culture supernatant was quantified with a Cytotoxicity Detection Kit (Roche Applied Science, Penzberg, Germany), according to the supplied instructions. LDH release is expressed as a percentage of the total content determined after lysing an equal amount of cells with 1% Triton X-100.

### Flow Cytometry

Splenocytes (6.0 × 10^6^ cells/well) were stimulated with ConA (5 μg/mL) in a 6-well plate in RPMI 1640 medium containing 10% heat-inactivated FBS, 100 units/ml of penicillin, and 100 μg/ml of streptomycin in an atmosphere of 5% CO_2_, 95% air at 37°C for 24 h. Splenocytes (5 × 10^5^ cells) were collected by centrifugation (4°C at 300 × *g*) and the supernatant was discarded. The cell pellet was washed twice with RPMI1640-based buffer and re-suspended in the same buffer. FITC rat anti-mouse CD4 (1 μL) (BD Pharmingen) and PE-conjugated anti-mouse CD25 (1 μL) (TOMBO) were added to 50 μL of the cell suspension and the mixture was incubated at room temperature for 30 min in the dark. The cells were then washed twice in RPMI1640-based buffer and immediately subjected to flow cytometry (FACSCalibur Flow Cytometer, Becton, Dickinson and Co., United States)

### Cell Proliferation Assay

Splenocyte proliferation was assessed by detection of bromodeoxyuridine (BrdU) uptake using the Cell Proliferation ELISA, BrdU (colorimetric) (Roche) according to the manufacturer’s protocol. Briefly, splenocytes (4 × 10^5^ cells) were seeded into 96-well plates in RPMI 1640 medium containing 10% heat-inactivated FBS, 100 units/ml of penicillin, and 100 μg/ml of streptomycin and labeled with BrdU (Roche) for 24 h. After centrifugation (10°C at 300 *g*), removal of the supernatant, and drying of the cells with a hair-dryer for about 15 min, 200 μL FixDenat reagent was added. The cells were incubated at room temperature for 30 min, then the FixDenat solution was washed out. The cells were incubated with peroxidase-conjugated anti-BrdU solution and the amount of incorporated BrdU was determined. The absorbance was measured with an ELISA plate reader (450 nm).

### Immunoblotting

Concanavalin A-stimulated murine splenocytes (1.0 × 10^7^ cells) were lysed, and the lysate was taken up in sample buffer (25% glycerin, 1% SDS, 62.5 mm Tris-Cl, 10 mm dithiothreitol) and boiled for 10 min. Aliquots of samples were analyzed by SDS–PAGE and transferred to a polyvinylidene difluoride membrane. The membrane was incubated at 4°C overnight with blocking buffer (10 mM Tris–HCl, 100 mM NaCl, 0.1% Tween 20, 1% BSA, pH 7.5), and then with rabbit mAb against phospho-ERK 1/2 (1:1000) or ERK 1/2 (1:1000) (Cell Signaling Technology, Beverly, MA, United States) at 4°C overnight. The membranes were washed four times with TBST, then incubated with goat horseradish peroxidase-conjugated anti-rabbit IgG Ab (1:20,000) (Cell Signaling Technology) for 1.5 h at room temperature. The membrane was further washed with TBST, and specific proteins were visualized by using ImmunoStar^®^LD (Wako). Western blotting detection reagents were from LI-COR, and bands were analyzed with Image Studio 4.0 for C-DiGit Scanner (LI-COR).

### Measurement of IL-2 mRNA Expression

Total RNA was extracted from lymphocytes, and first-strand cDNA was synthesized with PrimeScript Reverse Transcriptase (Takata Bio). The cDNA was used as a template for real-time PCR analysis: reactions were performed in a CFX Connect Real-Time System (Bio-Rad). RT2-qPCR^®^ primer assay for mouse IL-2 was purchased from Qiagen. Glyceraldehyde-3-phosphate dehydrogenase mRNA was determined as a positive control. Each sample was assayed in a 20 μL amplification reaction mixture, containing cDNA, primers and 2× KAPA SYBR^®^ FAST qPCR Master Mix (Kapa Biosystems). The samples were incubated at 95°C 1 min, then amplification was carried out for 40 cycles (each cycle, 95°C for 3 s, annealing at 60°C 30 s), followed by incubation at 95°C 1 min. Fluorescent products were detected at the last step of each cycle. The obtained values were within the linear range of the standard curve and were normalized to glyceraldehyde-3-phosphate dehydrogenase mRNA expression.

### Measurement of Intracellular IL-2 Protein

Splenocytes (1.0 × 10^7^ cells/mL) in a 6-well plate were stimulated with ConA (5 μg/mL) in RPMI 1640 medium containing 10% heat-inactivated FBS, 100 units/ml of penicillin, and 100 μg/ml of streptomycin in an atmosphere of 5% CO_2_, 95% air at 37°C. After incubation, the supernatant was discarded. The cells were lysed in 1 M HEPES-NaOH, 10% Triton/PBS, 0.5 M EDTA, pH 8.0, 1% protease inhibitor for 30 min at 4°C, followed by centrifugation at 300 × *g* (at 4°C). IL-2 protein in the culture supernatant was measured by means of ELISA, as described above.

### Treatment with ConA into Mice

BALB/c mice were injected intravenously (*i.v.*) with 400 μg of ConA in 100 μL PBS, or with 100 μL PBS as a control. After ConA treatment, blood samples were collected. They were centrifuged at 5,000 × *g* for 10 min at 4°C, and the separated serum was stored at -80°C until analysis. Serum concentrations of IL-2 and IL-6 were determined by means of ELISA as described above.

### Statistics

Results are expressed as mean ± SE. The statistical significance of differences between control and other groups was calculated by using Dunnett’s test with the Instat version 3.0 statistical package (GraphPad Software, San Diego, CA, United States). The criterion of significance was set at *P* < 0.05.

## Results

### Effect of ATP on Release of Inflammatory Cytokines from ConA-Stimulated Splenocytes

**FIGURE 1:**
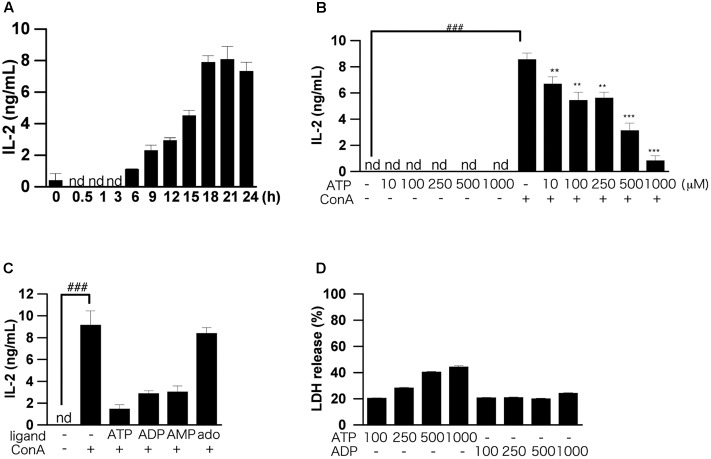
Effects of ATP on ConA-induced IL-2 production by splenic lymphocytes isolated from BALB/c mice. **(A)** Splenic lymphocytes were stimulated with 50 μg/mL ConA and incubated for the indicated times. The concentration of IL-2 released into the culture medium was measured by means of ELISA. **(B)** Lymphocytes were pre-incubated with various concentrations of ATP (10, 50, 100, 250, and 500 μM) for 30 min, and then stimulated with 50 μg/mL ConA for 24 h. **(C)** Lymphocytes were pre-incubated with 250 μM ATP, ADP, AMP, and adenosine (ado) for 30 min, and then stimulated with 50 μg/mL ConA for 24 h. **(D)** Cell damage after incubation of lymphocytes with ATP or ADP (100, 250, and 500 μM) for 1 h was measured by LDH assay. Each value represents the mean ± SE (*n* = 4). Significant difference between the vehicle control group and the ConA-treated group in the absence of ATP or other ligand is indicated by ^###^*P* < 0.001. Significant differences between the ConA-treated, ATP-untreated group and the ConA-treated, ATP-treated groups is indicated by ^∗∗∗^*P* < 0.001 and ^∗∗^*P* < 0.01. Each figure is representative of several independent experiments.

In our previous study, we found that production of IL-2, an inflammatory cytokine, in splenocytes of BALB/c mice was increased dose-dependently by ConA, and was maximum at 1–10 μg/mL ConA. Here, using ConA (5 μg/mL), we found that production of IL-2 increased time-dependently up to 24 h after ConA addition (**Figure 1A**). These results suggest that ConA directly induces IL-2 production in murine splenocytes. In the following experiments, assays of IL-2 secretion were all conducted at 24 h after stimulation with 5 μg/mL of ConA.

We first examined the dose dependence of the effect of exogenously added ATP on IL-2 production by splenocytes. Pretreatment with more than 100 μM ATP significantly suppressed production of IL-2 (**Figure 1B**). Next, we examined the effects of ATP, ADP, AMP, and adenosine on the ConA-induced IL-2 production. As shown in **Figure 1C**, treatment with ATP, ADP, and AMP suppressed the ConA-induced increase of IL-2 secretion at 24 h, although adenosine was ineffective. UTP and UDP did not suppress IL-2 production (data not shown).

**FIGURE 2:**
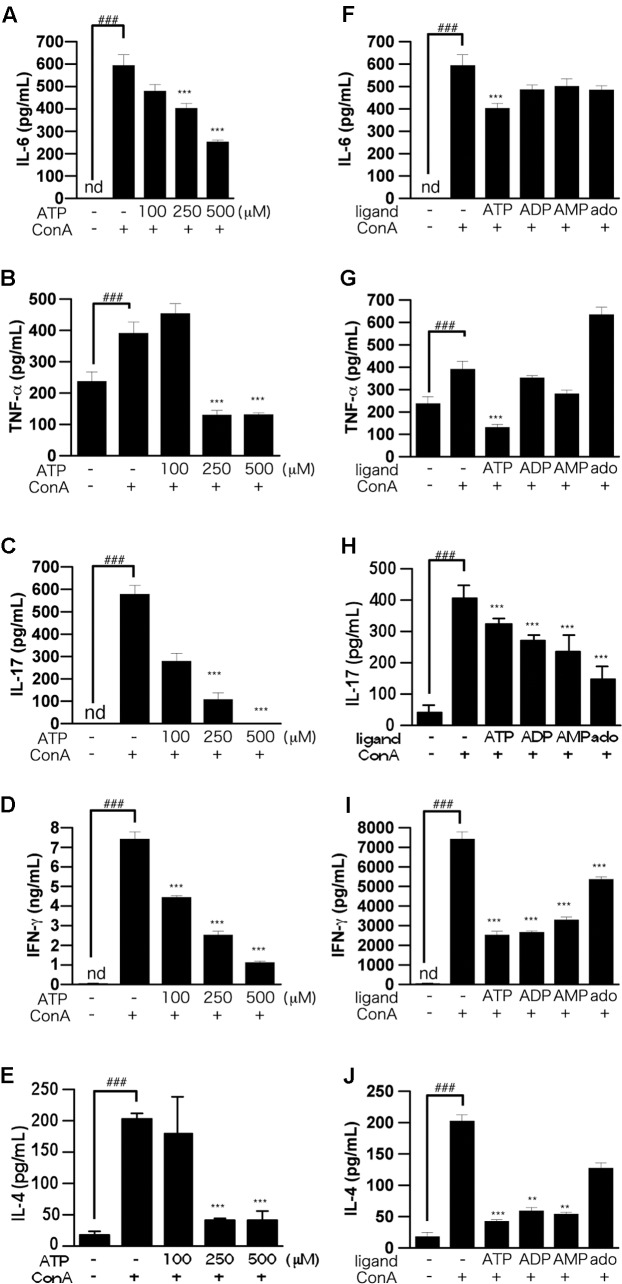
Effects of ATP on ConA-induced production of pro-inflammatory cytokines by splenic lymphocytes isolated from BALB/c mice. **(A–E)** Lymphocytes were pre-incubated with various concentrations of ATP (100, 250, or 500 μM), and then stimulated with 50 μg/mL ConA for 24 h. **(F–J)** Lymphocytes were pre-incubated with 250 μM ATP, ADP, AMP, or adenosine, and then stimulated with 50 μg/mL ConA for 24 h. Concentrations of IL-6 **(A,F)**, TNF-α **(B,G)**, IL-17 **(C,H)**, IFN-γ **(D,I)**, or IL-4 **(E,J)** in the culture medium were measured by ELISA. Each value represents the mean ± SE (*n* = 4). Significant difference between the vehicle control group and the ConA-treated group in the absence of ATP or other ligand is indicated by ^###^*P* < 0.001. Significant differences between the ConA-treated, ATP-untreated group and the ConA-treated, ATP (or other ligand)-treated groups is indicated by ^∗∗∗^*P* < 0.001 and ^∗∗^*P* < 0.01. Each figure is representative of several independent experiments.

The results of LDH assays showed that ATP concentration-dependently induced cell death, whereas ADP was not cytotoxic (**Figure 1D**). That is, the suppression of IL-2 production by 250 μM ATP might not be due to cytotoxicity mediated by activation of the P2X7 receptor (Tsukimoto et al., 2006). Also, production of IL-6, TNF-α, IL-17, IFN-γ, and IL-4, as well as IL-2, by ConA-stimulated lymphocytes was suppressed by ATP treatment (**Figures 2A–J**).

### Antagonists of Purinergic Receptors Did Not Block ATP-Induced Suppression of T Cell Activation

**FIGURE 3:**
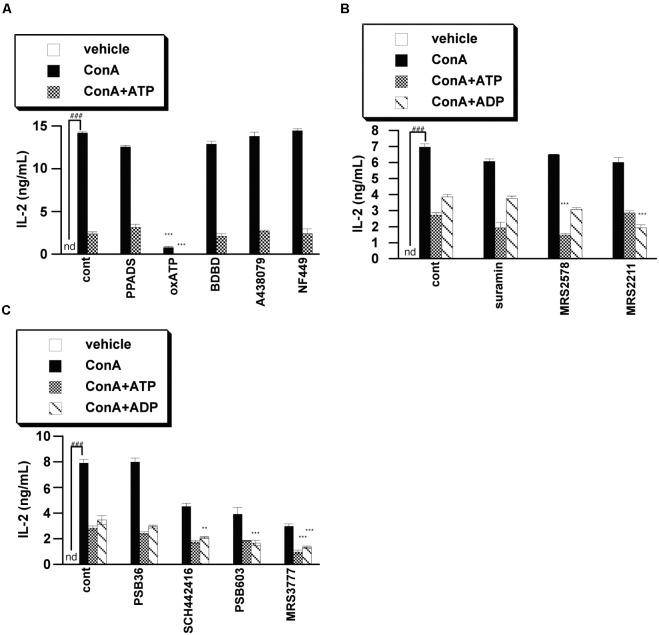
Effects of purinergic receptor antagonists on IL-2 secretion from activated splenic lymphocytes. Lymphocytes were pre-incubated with 250 μM ATP or ADP and with a P2X receptor inhibitor [PPADS (100 μM), oxATP (300 μM), BDBD (10 μM), A438079 (50 μM), NF449 (10 μM)] **(A)**, P2Y receptor inhibitor [suramin (100 μM), MRS2578 (10 μM), MRS2211 (100 μM)] **(B)**, or adenosine receptor inhibitor PSB36 (5 μM), SCH442416 (10 μM), PSB603 (10 μM), or MRS3777(10 μM) **(C)**, and then stimulated with 50 μg/mL ConA for 24 h. Concentrations of IL-2 in the culture medium were measured by ELISA. Significant difference between the vehicle control group and the ConA-treated group in the absence of ligand and inhibitor is indicated by ^###^*P* < 0.001. Significant differences between ConA-treated, inhibitor-untreated control (cont) groups and the corresponding inhibitor-treated groups are indicated by ^∗∗∗^*P* < 0.001 and ^∗∗^*P* < 0.01. Each value represents the mean ± SE (*n* = 4). Each figure is representative of several independent experiments.

We next examined whether purinergic receptors are involved in the ATP-induced suppression of IL-2 release by using of a variety of purinergic receptor antagonists. We found that antagonists of P2X receptors, including P2X7 receptor, did not block ATP-induced suppression of IL-2 release, suggesting that P2X receptors are not involved in the effect of ATP. On the other hand, P2Y6 antagonists (MRS2578), P2Y13 antagonists (MRS2211) and A3 antagonists (MRS3777) enhanced the suppression of IL-2 release by ATP or ADP, indicating that activation of these receptors by ATP contributes to the induction of IL-2 release, rather than inhibition of IL-2 release (**Figures 3A–C**). It seems likely that the suppression of IL-2 release from lymphocytes by ATP might be mediated by factor(s) other than purinergic receptors.

### ATP Treatment Suppressed CD25 Expression and Cell Proliferation after ConA Stimulation

**FIGURE 4:**
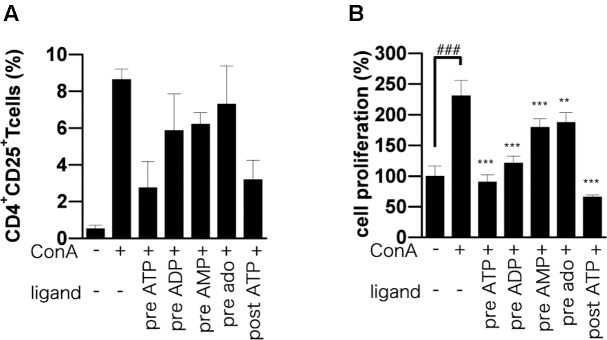
Effect of adenine nucleotides on expression of CD25 and proliferation of BrdU-labeled cells. Lymphocytes were pre-incubated for 30 min with 250 μM ATP, ADP, AMP, or adenosine before ConA treatment or post-incubated with ATP at 30 min after ConA treatment. **(A)** Cells were incubated for 24 h with ConA. The lymphocytes were stained with PE-conjugated anti-CD25 mAb and FITC-conjugated anti-CD4 mAb. The percentage of CD4^+^ CD25^+^ cells was analyzed by flow cytometry. **(B)** Cells were incubated for 21 h with ConA. Lymphocyte proliferation was determined by BrdU cell proliferation assay. Each value represents the mean ± SE (*n* = 4). Significant difference between the vehicle control group and the ConA-treated group in the absence of ATP or other ligand is indicated by ^###^*P* < 0.001. Significant differences between ConA-treated groups without ligand treatment and the corresponding groups given the indicated ligand treatment are indicated by ^∗∗∗^*P* < 0.001 and ^∗∗^*P* < 0.01. **(A)** Represents three independent experiments and **(B)** represents two independent experiments.

T cells up-regulate activation markers such as CD25 during T cell activation (Malek and Bayer, 2004), and activation of IL-2 receptor by IL-2 triggers a signaling cascade leading to T cell proliferation and further IL-2 production (Kim and Leonard, 2002). As expected, we found that CD25-positive CD4 T cells in ConA-stimulated lymphocytes were significantly reduced by adenine nucleotides (**Figure 4A**). To investigate the effect of ATP on T cell proliferation, we employed BrdU incorporation assay. The incorporation of BrdU into ATP-treated lymphocytes was significantly lower than that into untreated lymphocytes (**Figure 4B**).

### Post-treatment with ATP Also Suppressed IL-2 Release from T Cells Activated by ConA or TCR

**FIGURE 5:**
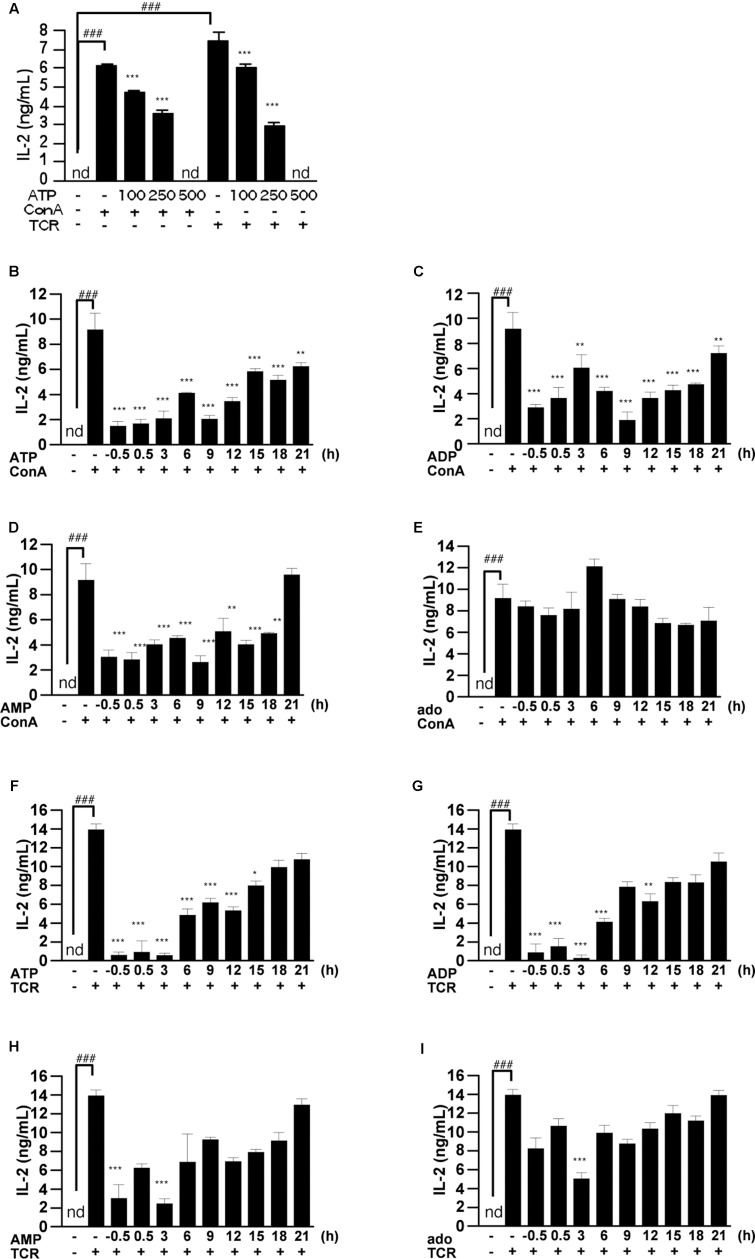
Effect of ATP, ADP, AMP, and adenosine on already activated T cells. **(A)** Lymphocytes were pre-incubated with ATP (100, 250, and 500 μM), and then stimulated with 50 μg/mL ConA or with plate-bound anti CD3ε mAb and soluble anti CD28 mAb for 24 h. Concentrations of IL-2 in the culture medium were measured by ELISA. **(B–I)** Lymphocytes were post-treated with ATP, ADP, AMP, and adenosine at the indicated time points after treatment with 50 μg/mL ConA **(B–E)** or with plate-bound anti CD3ε mAb and soluble anti CD28 mAb **(F–I)**. Cells were incubated for 24 h with ConA or the antibodies. Concentrations of IL-2 in the culture medium were measured by means of ELISA. Each value represents the mean ± SE (*n* = 4). Significant difference between the vehicle control group or the vehicle plus TCR group and the corresponding ConA-treated group in the absence of ligand is indicated by ^###^*P* < 0.001. Significant differences between ConA-treated groups without ligand treatment and the corresponding groups given the indicated ligand treatment are indicated by ^∗∗∗^*P* < 0.001 and ^∗∗^*P* < 0.01. Each figure is representative of several independent experiments.

To confirm whether the suppressive effect of ATP is dependent on the nature of the T cell activation stimulus, we examined IL-2 release from TCR-activated splenic lymphocytes. As shown in **Figure 5A**, treatment with ATP suppressed the increase of IL-2 secretion at 24 h after stimulation of splenocytes with TCR or ConA. To clarify whether ATP is also effective on prestimulated T cells, we treated splenocytes with adenine nucleotides after ConA stimulation. They proved effective in suppressing IL-2 release when applied up to 9 h after ConA or TCR stimulation (**Figures 5B–I**).

### Effect of ATP on IL-2 mRNA Expression and the ERK1/2 Pathway in Activated T Cells

**FIGURE 6:**
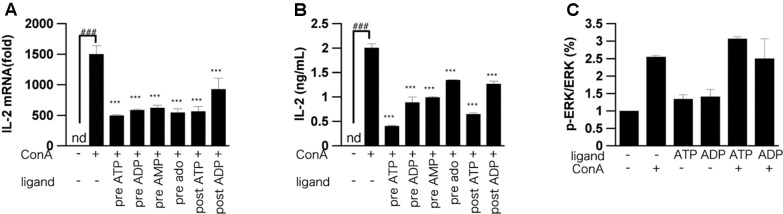
Effect of ATP, ADP, AMP and adenosine on IL-2 expression. Lymphocytes were pre-incubated or post-incubated with ATP, ADP, AMP, or adenosine, and then stimulated with 50 μg/mL ConA for 3 h **(A)**, 6 h **(B)**, or 5 min **(C)**. **(A)** IL-2 mRNA level in lymphocytes was measured by real-time RT-PCR analysis. **(B)**, Intracellular protein level of IL-2 was measured by means of ELISA. **(C)** Lymphocytes were lyzed and analyzed by western blotting for phospho-ERK1/2 and total ERK1/2. Each value represents the mean ± SE (*n* = 4). Significant difference between the vehicle control group and the ConA-treated group in the absence of ATP and ADP is indicated by ^###^*P* < 0.001. Significant differences between ConA-treated groups without ligand treatment and the corresponding groups given the indicated ligand treatment are indicated by ^∗∗∗^*P* < 0.001. **(A,B)** Represent two independent experiments and **(C)** represents three independent experiments.

IL-2 mRNA was increased in murine lymphocytes 3 h after ConA stimulation. We examined whether ATP and their metabolites affected this increase of IL-2 mRNA. Indeed, ATP or ADP significantly suppressed the increase of IL-2 mRNA (**Figure 6A**). However, the suppressive effect of AMP or adenosine was less than that of ATP or ADP. We also measured the intracellular IL-2 protein level in lymphocytes by ELISA. The IL-2 protein level was decreased by treatment with adenine nucleotides (**Figure 6B**). In order to investigate the signaling pathway involved into suppression of ConA-induced IL-2 release by ATP, phosphorylation of ERK were measured (**Figure 6C**). ATP did not affect the ConA-evoked phosphorylation of ERK1/2.

### Inhibitory Effect of ATP and ADP Analogs on IL-2 Release

**FIGURE 7:**
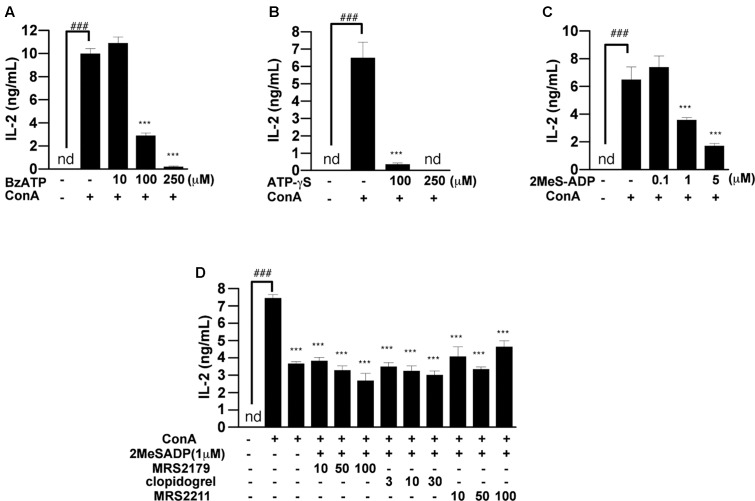
Effects of non-hydrolyzable ATP and ADP analog on ConA-induced IL-2 production. **(A)** Lymphocytes were pre-incubated with various concentrations of Bz-ATP **(A)**, ATP-γS **(B)**, and 2-MeS-ADP **(C)**. **(D)** Lymphocytes were pre-incubated with 1 μM 2-MeS-ADP and with MRS2179, clopidogrel, or MRS2211. Then, the lymphocytes were stimulated with 50 μg/mL ConA for 24 h. Concentrations of IL-2 in the culture medium were measured by means of ELISA. Each value represents the mean ± SE (*n* = 4). Significant difference between the vehicle control group and the ConA-treated group in the absence of any ligand is indicated by ^###^*P* < 0.001. Significant differences between the ConA-treated group without any ligand treatment and the corresponding ConA-treated groups given the indicated ligand treatment are indicated by ^∗∗∗^*P* < 0.001. Each figure is representative of several experiments.

To investigate what is the largest contributor to suppression of IL-2 release among ATP and its metabolites, we examined the effects of non-hydrolyzable ATP and ADP analogs (ATP-γS, BzATP, and 2-MeS-ADP) on IL-2 production. Bz-ATP and ATP-γS suppressed the ConA-induced increase of IL-2 secretion at 24 h, like ATP (**Figures 7A,B**). However, the reason why BzATP suppressed IL-2 release is presumably that BzATP-induced activation of the P2X7 receptor resulted in induction of cell death. On the other hand, the addition of 2-MeS-ADP had a potent inhibitory effect on IL-2 production (**Figure 7C**), suggesting that ADP is important for suppression of T cell activation. 2-MeS-ADP is known to have high affinities for the P2Y1, P2Y12, P2Y13 receptors (Macfarlane et al., 1983; Sak and Webb, 2002; Zhang et al., 2002). We therefore examined the effects of P2Y1, P2Y12, and P2Y13 receptor antagonists (MRS2179, clopidogrel, and MRS2211, respectively) on 2-MeS-ADP-induced suppression of IL-2 release. However, these antagonists had no effect (**Figure 7D**).

### Intravenous Administration of ATP Reduced Serum Pro-inflammatory Cytokine Levels

**FIGURE 8:**
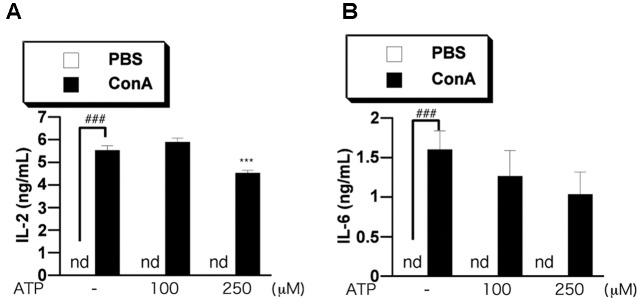
Treatment with ATP attenuates ConA-induced cytokine production *in vivo*. BALB/c mice (*n* = 5/group) were pretreated with PBS or ATP (100 or 250 μM) at 2 h before treatment with ConA. Mice were injected with ConA (400 μg of ConA in 100 μL PBS) via the tail vein. Serum samples were collected at 3 h **(A,B)** after the ConA injection. Serum IL-2 **(A)** and IL-6 **(B)** concentrations were determined as described in the text. Significant difference between the vehicle control group and the ConA-treated group in the absence of any ligand is indicated by ^###^*P* < 0.001. Significant differences between the ConA-treated group without any ligand treatment and the corresponding ConA-treated groups given the indicated ligand treatment are indicated by ^∗∗∗^*P* < 0.001.

Finally, we investigated the effect of ATP on the elevation of cytokines in blood of ConA-treated mice. We measured the levels of IL-2 and IL-6 in serum by ELISA. We previously confirmed that the levels of IL-2 and IL-6 were increased at 3–12 h after ConA injection, but then decreased until 24 h (data not shown). We examined the effect of ATP on the increase of proinflammatory cytokines in serum at 3 h after ConA injection into BALB/c mice. Pretreatment with 250 μM ATP suppressed the increase of serum IL-2 and tended to suppress IL-6 (**Figures 8A,B**), suggesting that ATP would suppress activation of immune cells including T cells *in vivo*.

## Discussion

T cells are activated and release inflammatory cytokines including IL-2 in response to stimulation with ConA. We found that the IL-2 release from ConA-activated T cells was suppressed by treatment with ATP. Specifically, we found that pretreatment with more than 100 μM ATP significantly and dose-dependently suppressed production of IL-2 by ConA-treated splenic lymphocytes of BALB/c mice. Further, the suppression of IL-2 production by 250 μM ATP did not appear to be due to cytotoxicity mediated by activation of P2X7 receptor (Di Virgilio et al., 1998; Tsukimoto et al., 2006). Our data show that ATP also suppressed the ConA-induced release of other cytokines (IL-6, IL-17, TNF-α, IFN-γ, and IL-4) from T cells.

Previous studies have demonstrated that P2X, P2Y, and adenosine receptors play important roles in the regulation of T cell activation (Lappas et al., 2005; Yip et al., 2007; Woehrle et al., 2010). We pharmacologically investigated the contribution of P2 receptors to the suppression of T cell activation, in order to determine whether purinergic receptors are involved in the suppression of IL-2 release. We found that antagonists of P2X receptors did not block ATP-induced suppression of IL-2 release, suggesting that P2X receptors were not involved in the effect of ATP. However, P2Y6 antagonist (MRS2578), P2Y13 antagonist (MRS2211) and A3 antagonist (MRS3777) strongly suppressed IL-2 release upon co-treatment with ATP or ADP. That is, we found that activation of these receptors by ATP contributes to induction of IL-2 release, rather than inhibition of IL-2 release. Thus, suppression of IL-2 release from lymphocytes by ATP might be mediated by other factor(s).

When T cells are activated, they express CD25 and produce IL-2, both of which are involved in T cell growth and proliferation (Morgan et al., 1976). Our results show that DNA replication monitored in terms of BrdU incorporation was greatly reduced by pretreatment with ATP and ADP, compared with AMP and adenosine. We also confirmed that ConA stimulation increased CD25 expression in T cells and that ATP and its metabolites suppressed the ConA-induced CD25 expression. In other words, it appears that ATP and ADP can suppress T cell proliferation and activation.

Pretreatment with ATP also suppressed the TCR-induced increase of IL-2 secretion at 24 h, as was the case for ConA stimulation. Even when ATP, ADP, AMP, and adenosine were added after ConA or TCR stimulation, ATP and ADP were effective in suppressing IL-2 release. In other words, ATP and ADP might have inhibitory functions on already activated T cells. Furthermore, pretreatment with ATP and ADP caused a marked reduction of ConA-induced IL-2 mRNA expression and intracellular protein levels. In order to investigate which intracellular signals are involved in the suppression of ConA-induced IL-2 release by ATP treatment, we measured phosphorylation of ERK1/2. Responses to stimuli such as TCR are mediated by MAPK, and MAPKs are involved in T cell activation and proliferation by activating various downstream factors. MAPKs play important roles in T cell activation, proliferation, and differentiation into Th1 or Th2 (Dong et al., 2002; Huang and Wange, 2004). Among MAPKs, ERK consists of two species, ERK 1 and 2, with molecular weights of 42 and 44 kDa, respectively. No significant suppression of ERK1/2 phosphorylation by ATP was observed after ConA stimulation in T cells, suggesting that ATP may affect pathways other than ERK1/2.

The inhibitory effect of ATP on ConA-induced IL-2 release was mimicked by non-hydrolyzable ATP and ADP analogs, ATP-γ-S, BzATP, and 2-MeS-ADP. The results of pretreatment with ATP-γS and BzATP were similar to those of pretreatment with ATP. But, since BzATP is known to be a P2X7 receptor agonist, it seems likely that the cell death induced by 250 μM BzATP was due to activation of P2X7 receptor. Since the addition of 2-MeS-ADP had a potent inhibitory effect on the production of IL-2, ADP seems to have an important role in the suppression of T cell activation. Pre-treatment with P2Y1, P2Y12, and P2Y13 antagonists before treatment with 2-MeS-ADP did not cause any significant change of IL-2 production compared with 2-MeS-ADP treatment alone. These results suggest that ADP may have a novel immunosuppressive effect, not mediated by P2Y1, P2Y12, or P2Y13 receptors.

There is some evidence that guanosine administration reduces brain damage and has an anti-inflammatory action (Ciccarelli et al., 2001), possibly via cAMP accumulation and activation of PI3K/Akt. It has also been reported that addition of deoxyguanosine triphosphate (dGTP), guanosine triphosphate (GTP) and guanosine inhibits T-cell proliferation, though the mechanisms involved remain unknown (Weiler et al., 2016). Thus, it is possible that new receptors activated by GDPs or ATP might be involved in the effects observed in the present study.

T cell activation plays an important role in autoimmune inflammatory diseases, such as rheumatoid arthritis, multiple sclerosis, and autoimmune hepatitis. Currently, adrenocortical hormone and immunosuppressants are mainly used for treatment of autoimmune diseases. However, adrenocortical hormone therapy causes serious side effects such as weight gain, osteoporosis, weakness of the skin and hair, abuse, diabetes, hypertension, cataracts, glaucoma, anxiety, and confusion. Immunosuppressive agents also cause serious side effects such as decreased leucocytes, nausea, vomiting, liver disorder, pancreatitis, and promotion of cancer growth. Therefore, a new treatment approach is urgently needed. Our present findings suggested that ATP or ADP might be useful for this purpose. Our preliminary data implicates that treatment with ATP tended to suppress the elevations of serum liver damage markers (glutamic oxaloacetic transaminase and glutamic pyruvic transaminase levels) in ConA-treated mice which is known as an autoimmune hepatitis model (Tiegs et al., 1992) (data not shown), suggesting that pretreatment with ATP might attenuate the acute liver damage, though the effect of ATP on actual liver damage should be carefully evaluated by liver histology in the mice in future study. ATP might at least have potential as a supplemental drug in the treatment of immune diseases, possibly allowing a reduction in the administered dose of the main treatment agent(s) and thereby potentially reducing the incidence or severity of side effects. Since ATP is an endogenous energy source, it should be safe to use as a supplemental treatment, though further studies would be needed to confirm its efficacy, and to optimize treatment concentration and schedule. Also, the target molecule for this action of adenine nucleotides is still unknown, and it will be important to identify it.

## Author Contributions

YS contributed to the acquisition of data for this work. MT contributed to the conception and design of the work. Both YS and MT contributed to the analysis and interpretation of data for the work, and wrote the paper.

## Conflict of Interest Statement

The authors declare that the research was conducted in the absence of any commercial or financial relationships that could be construed as a potential conflict of interest.

## Abbreviations

2-MeS-ADP: 2-(methylthio)adenosine 5′-diphosphate trisodium salt hydrate
ATP-γS: adenosine 5′-O-(3-thiotriphosphate)
BrdU: 5-bromodeoxyuridine
BzATP: 2′(3′)-O-(4-benzoylbenzoyl)adenosine 5′-triphosphate triethylammonium salt
ConA: concanavalin A
ERK: extracellular signal-regulated kinase
IL: interleukin
LDH: lactate dehydrogenase
mAb: monoclonal antibody
MAPK: mitogen-activated protein kinase
TCR: T cell receptor

## References

[B1] Burnstock G. (2009). Purinergic signalling: past, present and future. *Braz. J. Med. Biol. Res.* 42 3–8. 18853040 10.1590/s0100-879x2008005000037

[B2] Burnstock G. Boeynaems J. (2014). Purinergic signalling and immune cells. *Purinergic Signal.* 10 529–564. 10.1007/s11302-014-9427-2 25352330 PMC4272370

[B3] Chused T. M. Apasov S. Sitkovsky M. (1996). Murine T lymphocytes modulate activity of an ATP-activated P2Z-type purinoceptor during differentiation. *J. Immunol.* 157 1371–1380. 8759716

[B4] Ciccarelli R. Ballerini P. Sabatino G. Rathbone M. P. D’Onofrio M. Caciagli F. (2001). Involvement of astrocytes in purine-mediated reparative processes in the brain. *Int. J. Dev. Neurosci.* 19 395–414. 10.1016/S0736-5748(00)00084-8 11378300

[B5] Di Virgilio F. Chiozzi P. Falzoni S. Ferrari D. Sanz J. M. Venketaraman V. (1998). Cytolytic P2X purinoceptors. *Cell Death Differ.* 5 191–199. 10.1038/sj.cdd.4400341 10200464

[B6] Dong C. Davis R. J. Flavell R. A. (2002). MAP kinases in the immune response. *Annu. Rev. Immunol.* 20 55–72. 10.1146/annurev.immunol.20.091301.131133 11861597

[B7] Fischer A. M. Katayama C. D. Pagès G. Pouysségur J. Hedrick S. M. Adachi S. (2005). The role of erk1 and erk2 in multiple stages of T cell development. *Immunity* 23 431–443. 10.1016/j.immuni.2005.08.013 16226508

[B8] Gallo E. M. Canté-Barrett K. Crabtree G. R. (2006). Lymphocyte calcium signaling from membrane to nucleus. *Nat. Immunol.* 7 25–32. 10.1038/ni1295 16357855

[B9] Gong Q. Cheng A. M. Akk A. M. Alberola-Ila J. Gong G. Pawson T. (2001). Disruption of T cell signaling networks and development by Grb2 haploid insufficiency. *Nat. Immunol.* 2 29–36. 10.1038/83134 11135575

[B10] Huang Y. Wange R. L. (2004). T cell receptor signaling: beyond complex complexes. *J. Biol. Chem.* 279 28827–28830. 10.1074/jbc.R400012200 15084594

[B11] Kim H.-P. Leonard W. J. (2002). The basis for TCR-mediated regulation of the IL-2 receptor alpha chain gene: role of widely separated regulatory elements. *EMBO J.* 21 3051–3059. 10.1093/emboj/cdf321 12065418 PMC126074

[B12] Kondo M. Takeshita T. Ishii N. Nakamura M. Watanabe S. Arai K. (1993). Sharing of the interleukin-2 (IL-2) receptor gamma chain between receptors for IL-2 and IL-4. *Science* 262 1874–1877. 10.1126/science.8266076 8266076

[B13] Lappas C. M. Rieger J. M. Linden J. (2005). A2A Adenosine receptor induction inhibits IFN-γ production in murine CD4+ T cells. *J. Immunol.* 174 1073–1080. 10.4049/jimmunol.174.2.1073 15634932

[B14] Lei H.-Y. Chang C.-P. (2009). Lectin of concanavalin A as an anti-hepatoma therapeutic agent. *J. Biomed. Sci.* 16:10. 10.1186/1423-0127-16-10 19272170 PMC2644972

[B15] Lin J. X. Leonard W. J. (1997). Signaling from the IL-2 receptor to the nucleus. *Cytokine Growth Factor Rev.* 8 313–332. 10.1016/S1359-6101(97)00021-X 9620644

[B16] Macfarlane D. E. Srivastava P. C. Mills D. C. (1983). 2-Methylthioadenosine[beta-32P]diphosphate. An agonist and radioligand for the receptor that inhibits the accumulation of cyclic AMP in intact blood platelets. *J. Clin. Invest.* 71 420–428. 10.1172/JCI110786 6298277 PMC436889

[B17] Malek T. R. Bayer A. L. (2004). Tolerance, not immunity, crucially depends on IL-2. *Nat. Rev. Immunol.* 4 665–674. 10.1038/nri1435 15343366

[B18] Marshall C. J. Alessi D. Saito Y. Campbell D. Cohen P. Sithanandam G. (1995). Specificity of receptor tyrosine kinase signaling: transient versus sustained extracellular signal-regulated kinase activation. *Cell* 80 179–185. 10.1016/0092-8674(95)90401-8 7834738

[B19] Morgan D. A. Ruscetti F. W. Gallo R. (1976). Selective in vitro growth of T lymphocytes from normal human bone marrows. *Science* 193 1007–1008. 10.1126/science.181845 181845

[B20] Nelson B. H. Lord J. D. Greenberg P. D. (1994). Cytoplasmic domains of the interleukin-2 receptor β and γ chains mediate the signal for T-cell proliferation. *Nature* 369 333–336. 10.1038/369333a0 7514277

[B21] Oh-hora M. (2009). Calcium signaling in the development and function of T-lineage cells. *Immunol. Rev.* 231 210–224. 10.1111/j.1600-065X.2009.00819.x 19754899

[B22] Rincon M. Conze D. Weiss L. Diehl N. L. Fortner K. A. Yang D. (2000a). Do T cells care about the mitogen-activated protein kinase signalling pathways? *Immunol. Cell Biol.* 78 166–175. 10.1046/j.1440-1711.2000.00900.x 10762418

[B23] Rincon M. Flavell R. A. Davis R. A. (2000b). The JNK and P38 MAP kinase signaling pathways in T cell–mediated immune responses. *Free Radic. Biol. Med.* 28 1328–1337. 10.1016/S0891-5849(00)00219-7 10924852

[B24] Sak K. Webb T. E. (2002). A retrospective of recombinant P2Y receptor subtypes and their pharmacology. *Arch. Biochem. Biophys.* 397 131–136. 10.1006/abbi.2001.2616 11747319

[B25] Sass G. Heinlein S. Agli A. Bang R. Schümann J. Tiegs G. (2002). Cytokine expression in three mouse models of experimental hepatitis. *Cytokine* 19 115–120. 10.1006/cyto.2002.1948 12242077

[B26] Schenk U. Westendorf A. M. Radaelli E. Casati A. Ferro M. Fumagalli M. (2008). Purinergic control of T cell activation by ATP released through pannexin-1 hemichannels. *Sci. Signal.* 1:ra6. 10.1126/scisignal.1160583 18827222

[B27] Tiegs G. Hentschel J. Wendel A. (1992). A T cell-dependent experimental liver injury in mice inducible by concanavalin A. *J. Clin. Invest.* 90 196–203. 10.1172/JCI115836 1634608 PMC443081

[B28] Tokunaga A. Tsukimoto M. Harada H. Moriyama Y. Kojima S. (2010). Involvement of SLC17A9-dependent vesicular exocytosis in the mechanism of ATP release during T cell activation. *J. Biol. Chem.* 285 17406–17416. 10.1074/jbc.M110.112417 20382737 PMC2878504

[B29] Trabanelli S. Ocadlíková D. Gulinelli S. Curti A. Salvestrini V. Vieira R. (2012). Extracellular ATP exerts opposite effects on activated and regulatory CD4^+^ T cells via purinergic P2 receptor activation. *J. Immunol.* 189 1303–1310. 10.4049/jimmunol.1103800 22753942

[B30] Tsukimoto M. Maehata M. Harada H. Ikari A. Takagi K. Degawa M. (2006). P2X7 receptor-dependent cell death is modulated during murine T cell maturation and mediated by dual signaling pathways. *J. Immunol.* 177 2842–2850. 10.4049/jimmunol.177.5.2842 16920919

[B31] Tsukimoto M. Tokunaga A. Harada H. Kojima S. (2009). Blockade of murine T cell activation by antagonists of P2Y6 and P2X7 receptors. *Biochem. Biophys. Res. Commun.* 384 512–518. 10.1016/j.bbrc.2009.05.011 19426712

[B32] Weiler M. Schmetzer H. Braeu M. Buhmann R. (2016). Inhibitory effect of extracellular purine nucleotide and nucleoside concentrations on T cell proliferation. *Exp. Cell Res.* 349 1–14. 10.1016/j.yexcr.2016.05.017 27233214

[B33] Whitehurst C. E. Geppert T. D. (1996). MEK1 and the extracellular signal-regulated kinases are required for the stimulation of IL-2 gene transcription in T cells. *J. Immunol.* 156 1020–1029. 8557975

[B34] Woehrle T. Yip L. Elkhal A. Sumi Y. Chen Y. Yao Y. (2010). Pannexin-1 hemichannel-mediated ATP release together with P2X1 and P2X4 receptors regulate T-cell activation at the immune synapse. *Blood* 116 3475–3484. 10.1182/blood-2010-04-277707 20660288 PMC2981474

[B35] Yip L. Cheung C. W. Corriden R. Chen Y. Insel P. A. Junger W. G. (2007). Hypertonic stress regulates T-cell function by the opposing actions of extracellular adenosine triphosphate and adenosine. *Shock* 27 242–250. 10.1097/01.shk.0000245014.96419.3a 17304104

[B36] Yip L. Woehrle T. Corriden R. Hirsh M. Chen Y. Inoue Y. (2009). Autocrine regulation of T-cell activation by ATP release and P2X7 receptors. *FASEB J.* 23 1685–1693. 10.1096/fj.08-126458 19211924 PMC2718802

[B37] Zhang F. L. Luo L. Gustafson E. Palmer K. Qiao X. Fan X. (2002). P2Y(13): identification and characterization of a novel Galphai-coupled ADP receptor from human and mouse. *J. Pharmacol. Exp. Ther.* 301 705–713. 10.1124/jpet.301.2.705 11961076

[B38] Zhang Y. L. Dong C. (2005). MAP kinases in immune responses. *Cell. Mol. Immunol.* 2 20–27. 16212907

